# Dual Malignancies Discovered: A Rare Case of Malignant Peritoneal Epithelioid Mesothelioma and Lung Adenocarcinoma

**DOI:** 10.7759/cureus.59962

**Published:** 2024-05-09

**Authors:** Mark A Potesta, Emma Guld, Jennifer Laman

**Affiliations:** 1 Internal Medicine, Lake Erie College of Osteopathic Medicine, Bradenton, USA; 2 Pediatrics, Lake Erie College of Osteopathic Medicine, Bradenton, USA; 3 Family Medicine, Access Healthcare Physicians, Hudson, USA

**Keywords:** independent malignancies, immunohistochemistry staining, adenocarcinoma, immunocytochemistry, asbestos, malignant mesothelioma, mesothelioma, malignant peritoneal epithelioid mesothelioma, lung adenocarcinoma, malignant peritoneal mesothelioma

## Abstract

Clinicians diagnosing malignant peritoneal epithelioid mesothelioma (MPM or MPeM) have historically had challenges due to the low incidence of the disease, as well as the often vague symptomatology that patients present with. Newer advances in technology, specifically in immunocytochemistry, have provided a clearer path to diagnosis. Additionally, malignant mesotheliomas must be differentiated from carcinomas. This is done via histology, immunocytochemistry, as well as a careful incorporation of the patient’s clinical history. In this case, we present an asymptomatic 73-year-old non-smoker female with no past medical history of asbestos exposure. She was diagnosed with MPM following a routine abdominal hernia repair. Subsequent workup revealed a lung infiltrate that was successfully biopsied and resected, evidently found to be adenocarcinoma. A careful review of the resulting pathology, as well as the interpretation of immunocytochemistry, supported the notion that the patient had two independent malignant processes occurring at once. This case underscores the rarity of two similar, yet distinct cancers, as well as epidemiology, symptomatology, histology, immunocytochemistry, and prognosis.

## Introduction

Mesothelioma is a disease of the pleural lining, peritoneum, and pericardial cavities. There are three common variants associated with the disease such as epithelioid, sarcomatous or desmoplastic, and biphasic [1]. Histologically, epithelioid mesothelioma cells may be observed shedding into effusions in the form of sheets, clusters or morulae, papillae, and individual cells [1]. Diagnosing mesothelioma presents challenges as it must be differentiated from reactive mesothelial cells as well as from adenocarcinoma cells [1]. Historically, this has been a difficult diagnosis to make due to the minute discrepancies when contrasting the former examples. Recently developed advances in molecular, genetic, and immunocytochemistry have shed light on confirming diagnosis in specimens [1]. Confirmation of peritoneal mesothelioma is challenging due to presenting symptoms being vague, non-specific, or even asymptomatic [2]. The non-specific symptoms can be linked to the widespread dissemination of small tumors in the abdominal cavity [2]. If symptoms do manifest, it is most commonly in the form of abdominal girth [2]. Pleural mesotheliomas are the most common, with about 90% of patients having a primary diagnosis at this site [1]. Following pleural mesotheliomas, the next most common are peritoneal and pericardial at 6-10% [1]. Other locations, outside the three mentioned, are exceedingly rare. Epidemiologically, 3,000 patients are diagnosed with malignant mesothelioma each year, with malignant peritoneal mesothelioma (MPM) making up fewer than 500 of these cases [2]. The infrequent nature of this cancer, as well as historical difficulties in confirming diagnosis has made it challenging to study over the years. Additionally, MPM confined to the peritoneal cavity only adds to the rarity.

## Case presentation

**Figure 1:**
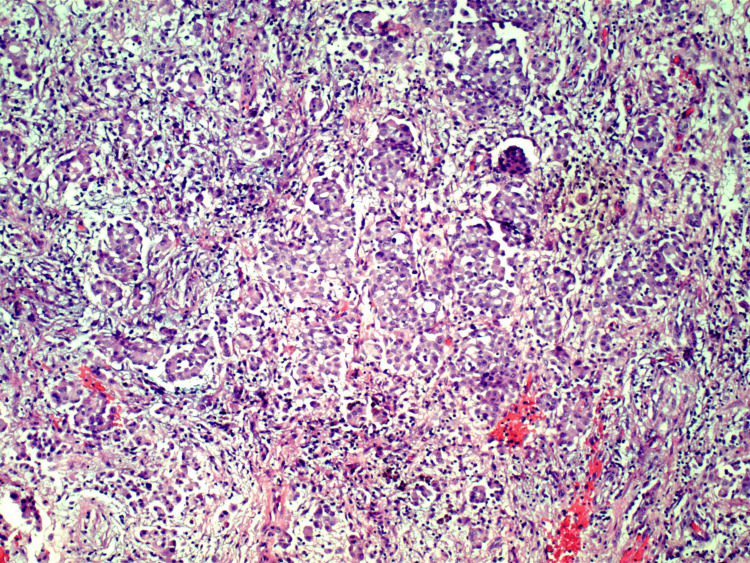
Peritoneal biopsy showing rare atypical epithelioid cells (10x magnification)

**Figure 2:**
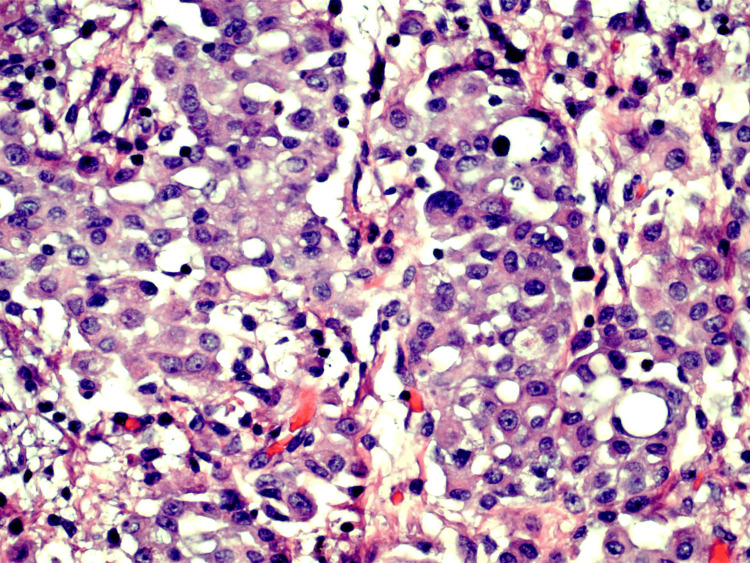
Peritoneal biopsy showing rare atypical epithelioid cells (40x magnification)

**Figure 3:**
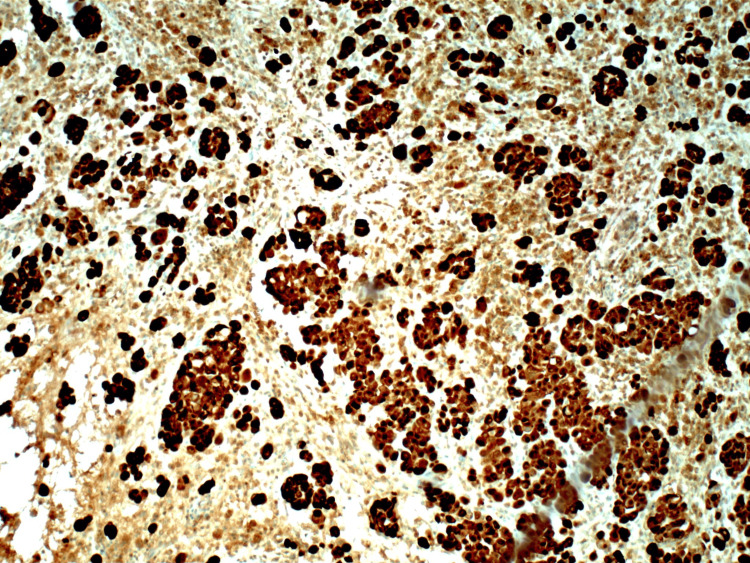
Peritoneal biopsy showing rare atypical epithelioid cells that stain positive for calretinin Highly sensitive stain for epithelioid malignant mesothelioma

**Figure 4:**
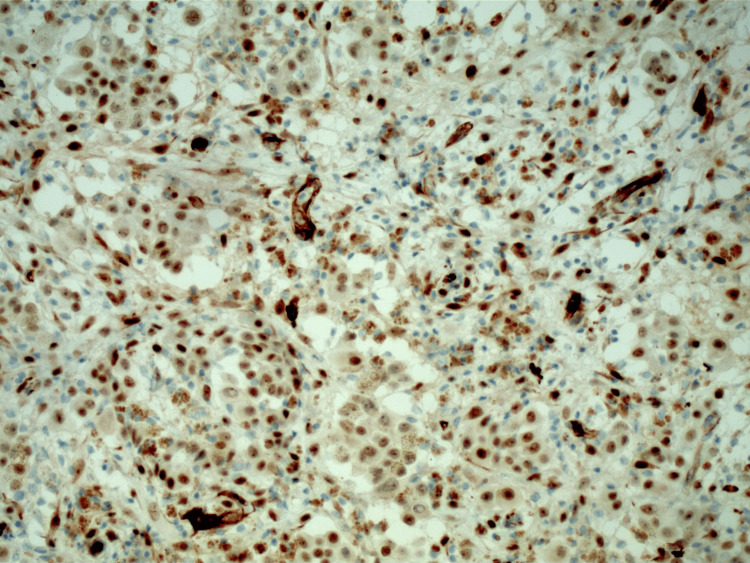
Peritoneal biopsy showing rare atypical epithelioid cells that stain positive for WT1 Highly sensitive stain for epithelioid malignant mesothelioma WT1: Wilms tumor gene 1

A 73-year-old non-smoker female with no history of asbestos exposure, or other chronic illnesses, presented with a diagnosis of MPM, discovered following routine umbilical hernia surgery. Her pathology report showed rare, atypical epithelioid cells that highlighted with Wilms tumor gene 1 (WT1), calretinin, pancytokeratin, cytokeratin (CK) 5/6 and negative for desmin, paired box gene 8 (PAX8), epithelial membrane antigen (​​​​​EMA), MOC31, BerEp4, and BRCA1-associated protein 1 (BAP1)* *(Figures 1-4)*.* Following pathology confirmation, the patient underwent a PET/CT that revealed a right upper lobe pleural mass without any other metabolic lesions. At this time, she received one cycle of carboplatin/pemetrexed along with a pulmonology referral for suspected pleural mesothelioma. She subsequently underwent therapeutic bronchoscopy with suctioning, right thoracoscopic wedge, thoracic lymphadenectomy, as well as right pleural biopsy. Ensuing pathology report returned invasive mixed mucinous and nonmucinous adenocarcinoma whose cells were positive for CK7, CK20, caudal type homeobox 2 (​​​​​​​CDX2), special AT-rich binding protein 2 ​​​​​​​(SATB2), and calretinin, while they were negative for thyroid transcription factor 1 ​​​​​​​(TTF1)*, *p40,PAX8,estrogen receptor​​​​​​​ (ER) and progesterone receptor​​​​​​​(PR),WT1, and D2-40. The patient’s pleural biopsy was negative for malignancy, thus evidence of pleural mesothelioma was not found. Her adenocarcinoma was staged T2aN0M1 and was able to be fully resected with clear margins. Now it was believed that the patient had two independent malignant processes, with the MPM not having a clear primary source. At this time, it was determined that a diagnostic laparoscopic procedure was necessary to reevaluate the diagnosis of MPM. Diagnostic laparoscopy re-confirmed the diagnosis and without a primary source that could be detected via PET/CT scan, as well as lung biopsy, the patient was deemed to be stage IV. Subsequently, the patient underwent four additional cycles of carboplatin/pemetrexed (totaling five) before cessation of chemotherapy in lieu of immunotherapy with nivolumab/ipilimumab. The patient tolerated both therapies well and most recent imaging studies following five rounds of chemo and two months of immunotherapy have shown stability in her disease process. This case highlights the rare nature of two independent malignant processes discovered following a routine surgical procedure in an otherwise asymptomatic patient with no historical risk factors.

## Discussion

As previously stated, one of the challenges of diagnosing MPM is differentiating it from adenocarcinoma. Using a combination of typical histological differences along with advances in immunocytochemistry has provided clarity. In the case of mesothelioma, relatively constant cytologic features used to aid in diagnosis include the presence of a single malignant mesothelial cell population, multinucleation, articulation between mesothelial cells, cell-in-cell arrangements, cytoplasmic vacuoles, peripheral blebs, cluster formation with knobby outlines, variable nuclear enlargement, prominent nucleoli, and cytoplasmic metachromasia [1]. The acinar formation is a characteristic of adenocarcinomas, not mesotheliomas [1]. While intercellular windows between mesothelial cells might occasionally resemble acini, adenocarcinoma typically lacks this characteristic feature [1]. Another way to differentiate mesothelial cells from adenocarcinoma is that in adenocarcinoma the cell groups retain a smooth contour, also called a “community border” [1]. This is opposed to the knobby outlines of the mesothelioma cells [1].

The high likelihood of two independent malignant processes occurring in this patient is discernible from histology as well as an understanding sensitivity and specificity of immunocytochemistry markers. While imperfect, an immuno-panel containing two mesothelial markers (WT1, calretinin, or CK 5/6) and two epithelial markers (MOC 3, BerEp4) can offer sufficient sensitivity and specificity [3]. Studies specific to determining malignancy can assist in the verification of the diagnosis of malignant mesothelioma once a mesothelial lineage is identified [3]. BAP1 loss (seen in this patient), CDKN2A homozygous deletion, and MTAP loss are highly specific markers of malignancy in malignant mesotheliomas and also attain appropriate sensitivity when applied in a diagnostic panel, such as in this case [3]. 

The patient’s peritoneal biopsy yielded rare, morphologically atypical epithelioid cells that highlighted with WT1, calretinin, pancytokeratin, CK 5/6 and negative for desmin, PAX8, EMA, MOC31, BerEp4, and BAP1 (Tables 1-4). WT1, calretinin, and CK 5/6 are 70-100%, 80-100%, and 51-100% sensitive, respectively, for MPM [3]. MOC31 and BerEp4 are epithelial markers that are 85-100% sensitive to lung adenocarcinoma, lung squamous cell carcinoma, breast carcinoma, and high-grade serous ovarian carcinoma [3]. The combination of the pathology findings from initial laparoscopy status/post hernia repair, findings from her diagnostic laparoscopy, as well as understanding the immunocytochemistry, we were able to effectively rule in MPM. Additionally, the patient presented with an elevated CA-125 tumor marker of 158.7. Preceding treatment, CA-125 was elevated in >50% of patients in multiple studies and was identified as a marker for the reduction of the presence of cancerous cells and as a monitor for recurrence of disease after initial treatments (Table 5) [2]. Similarly, soluble/serum mesothelin-related protein (SMRP) can also serve as an accurate marker [2].

**Table 1:** Sensitivities of patient's peritoneal biopsy diagnostic up-regulated markers EpMM: epithelioid malignant mesothelioma; SaMM: sarcomatous malignant mesothelioma

Present markers	EpMM	SaMM
Wilms tumor gene 1 (WT1)	70-100%	-
Calretinin	80-100%	50-60%
Pancytokeratin	~100%	-
Cytokeratin 5/6	51-100%	-

**Table 2:** Specificities of patient's peritoneal biopsy diagnostic up-regulated markers BC: breast carcinoma; HGSOC: high-grade serous ovarian carcinoma; LUAD: lung adenocarcinoma; LUSC: lung squamous cell carcinoma; SaMM: sarcomatous malignant mesothelioma; CwEM: carcinoma with epithelioid morphology

Present markers	LUAD	LUSC	BC	HGSOC	SaMM	CwEM
Wilms tumor gene 1 (WT1)	0%	0-2%	5-8%	~100%	-	-
Calretinin	0-10%	35-40%	13%	13-31%	10-60%	-
Pancytokeratin	-	-	-	-	-	100%
Cytokeratin 5/6	5%	95-100%	5%	25-31%	-	-

**Table 3:** Sensitivities of patient's peritoneal biopsy diagnostic down-regulated markers BC: breast carcinoma; EpMM: epithelioid malignant mesothelioma; HGSOC: high-grade serous ovarian carcinoma; LUAD: lung adenocarcinoma; LUSC: lung squamous cell carcinoma; MM: malignant mesothelioma; PM: peritoneal mesothelioma; PMM: pleural mesothelioma; SaMM: sarcomatous malignant mesothelioma

Present markers	LUAD	LUSC	BC	HGSOC	SaMM	PM	PMM	EpMM
Paired box gene 8 (PAX8)	-	-	-	93-100%	-	-	-	-
MOC31	85-100%	85-100%	85-100%	85-100%	-	-	-	-
BerEp4	85-100%	85-100%	85-100%	85-100%	-	-	-	-
BRCA1-associated protein 1 (BAP1)	-	-	-	-	0-22%	55-67%	50-65%	61-77%

**Table 4:** Specificities of patient's peritoneal biopsy diagnostic down-regulated markers EpMM: epithelioid malignant mesothelioma; PM: peritoneal mesothelioma; HGSOC: high-grade serous ovarian carcinoma; MM: malignant mesothelioma *Specific for malignancy of mesothelial origin and MM when comparing to similar diagnoses (i.e. adenocarcinoma and HGSOC)

Present markers	PM	EpMM
Paired box gene 8 (PAX8)	6-18%	-
MOC31	-	0-10%
BerEp4	-	0-10%
BRCA1-associated protein 1 (BAP1)	100%*	100%*

**Table 5:** Tumor markers

Tumor marker	Patient's value	Reference range
Carcinoembryonic antigen (CEA)	<1.7	0-2.9 ng/mL
Cancer antigen 125 (CA 125)	158.7	0-35 U/mL
Cancer antigen 19-9 (CA 19-9)	3.8	0-37 U/mL

In terms of the lung biopsy, the patient’s pathology report returned invasive mixed mucinous and nonmucinous adenocarcinoma whose cells were positive for CK7, CK20, CDX2, SATB2, and calretinin*,* while they were negative for TTF1, p40, PAX8, ER and PR, WT1, and D2-40 (Tables 6-7). While calretinin is 80-100% sensitive for MPM, it can also be up to 10% specific for lung adenocarcinoma, thus it should not be used as a standalone finding [3]. CK* *is a main cytoskeleton protein that is mainly distributed to epithelial cells and specific subtypes play a role in differentiating primary vs secondary lung adenocarcinoma [4]. The patient also tested negative both for WT1 as well as D2-40, which have a respective sensitivity for MPM of 70-100% and 80-100%. After wedge resection with clear margins of the afflicted lobe of the lung, a greater understanding of the disease was obtained. The combination of immunocytochemistry results, which showed the lung biopsy being negative for two markers with high sensitivity for MPM, and pathology demonstrating invasive mixed mucinous and nonmucinous adenocarcinoma, effectively ruled out malignant mesothelioma as the likely source of the lung cancer and instead indicated adenocarcinoma. This further supports the notion that the patient had two independent malignant processes occurring at once.

**Table 6:** Sensitivities of patient’s lung biopsy diagnostic down-regulated markers EpMM: epithelioid malignant mesothelioma; HGSOC: high-grade serous ovarian carcinoma

Present markers	HGSOC	EpMM
Paired box gene 8 (PAX8)	93-100%	-
Estrogen receptor (ER)	40-90%	-
Progesterone receptor (PR)	20-40%	-
Wilms tumor gene 1 (WT1)	-	70-100%
Podoplanin (D2-40)	-	80-100%

**Table 7:** Specificities of patient’s lung biopsy diagnostic down-regulated markers BC: breast carcinoma; HGSOC: high-grade serous ovarian carcinoma; LUAD: lung adenocarcinoma; LUSC: lung squamous cell carcinoma; PM: peritoneal mesothelioma Note: The pertinent up-regulated marker, calretinin, should not be used as a standalone finding due to high sensitivity for malignant peritoneal mesothelioma and ~10% specificity for adenocarcinoma

Present markers	LUAD	LUSC	BC	HGSOC	PM
Paired box gene 8 (PAX8)	-	-	-	-	6-18%
Estrogen receptor (ER)	-	-	-	-	0-2%
Progesterone receptor (PR)	-	-	-	-	0-7%
Wilms tumor gene 1 (WT1)	0%	0-2%	0-3%	15-65%	-
Podoplanin (D2-40)	0-3%	15-42%	0-3%	15-65%	-

A widely known link between asbestos exposure and mesothelioma exists, as first reported in 1960 [1]. However, this link is commonly associated with malignant mesothelioma of the pleura, not MPM. Overall, 80% of patients with pleural mesothelioma have a history of asbestos exposure, while only 50% of patients with MPM have a history of asbestos exposure [5]. Incidence of disease has been documented to be higher in men as historically they have had greater exposure to at-risk careers such as working in the naval, mining, textile, and construction industries [6]. The patient in this case has no history of occupational or environmental exposure. In terms of this patient’s demographic, a female non-smoker, adenocarcinoma of the lung is the most common subtype (93%) [7]. As the patient had a successful wedge resection of the lung with clear margins, no further treatment was indicated for her adenocarcinoma. 

Although all variants of MPM have poor prognosis, of the three most common subtypes, studies have shown that on average, patients with the epithelioid subtype, such as in this case, have the “longest” prognosis [2]. The survival was observed at 51.5 months for patients with the epithelioid or well-differentiated papillary/cystic subtype. Patients with the sarcomatoid and biphasic types displayed an average survival of 10.5 months [2]. The patient underwent five rounds of pemetrexed/cisplatin chemotherapy before switching to immunotherapy with nivolumab/ipilimumab. Pemetrexed-based chemotherapeutics are broadly used for patients who are not surgical candidates [2]. Studies have also shown that the combination of nivolumab/ipilimumab has significantly extended survival vs chemotherapy [8].

## Conclusions

MPM is a rare form of cancer that is often detected late and associated with poor prognosis. Challenges associated with diagnosing MPM are differentiation from adenocarcinoma, low incidence, and infrequency of early onset symptoms. Advancements in immunocytochemistry, histology, and genetics have led to increased accuracy of diagnosis and earlier detection. Although long-term prognosis is still suboptimal, revelations in both chemotherapy and immunotherapy treatment options have shown promise. The patient tolerated both chemotherapy and immunotherapies well and has shown stability in her disease process via imaging as well as clinically. In an evolving field, this case emphasizes the uncommon occurrence of two separate malignant conditions detected after a routine surgical procedure in a patient who was otherwise asymptomatic and lacked any known risk factors.
